# Experiences of users and family members on the care received at community mental health centers in Lima and Callao during the COVID-19 pandemic

**DOI:** 10.17843/rpmesp.2023.403.12717

**Published:** 2023-09-25

**Authors:** Noelia Cusihuaman-Lope, Ana L. Vilela-Estrada, Victoria Cavero, David Villarreal-Zegarra, Francisco Diez-Canseco

**Affiliations:** 1 CRONICAS Centro de Excelencia en Enfermedades Crónicas, Universidad Peruana Cayetano Heredia, Lima, Peru. Universidad Peruana Cayetano Heredia CRONICAS Centro de Excelencia en Enfermedades Crónicas Universidad Peruana Cayetano Heredia Lima Peru; 2 Instituto Peruano de Orientación Psicológica, Lima, Peru. Instituto Peruano de Orientación Psicológica Lima Peru

**Keywords:** Attention, Mental Health, Users, Caregivers, Community Mental Health Center

## Abstract

**Objective.:**

To understand the experiences of new and continuing users of Community Mental Health Centers (CMHC) of Lima and Callao, and their relatives, regarding the mental health care they received during the COVID-19 pandemic.

**Materials and methods.:**

Qualitative study conducted between September 2021 and February 2022, in which we interviewed 24 users and family members who interacted with the services provided by three CMHCs in Lima and one in Callao during the COVID-19 pandemic. We carried out a thematic analysis of the transcribed interviews.

**Results.:**

Participants perceived that the pandemic exacerbated the symptoms of people with mental health problems. During the pandemic, mental health care relied on the use of technology, mainly telephone calls, which were used to monitor the emotional state and pharmacological treatment of users, as well as to schedule and remember appointments. The users emphasized that frequent telephone calls made them feel accompanied and highlighted the commitment of the CMHC workers. Among the difficulties, they reported an increase in the demand for care, problems in accessing video calls, and low quality in virtual care.

**Conclusions.:**

COVID-19 had an emotional impact on people with mental health problems; in turn, CMHC services were affected by the type of care (face-to-face or virtual), resources, frequency, time and quality of care, finding limitations and benefits in the use of technology.

## INTRODUCTION

Prior to the COVID-19 pandemic, 20% of the Peruvian population had some type of mental disorder, and only 3 out of 10 residents of Lima and Callao who required mental health (MH) care received it ^1^. On March 15, 2020, the Peruvian government declared a State of National Emergency and implemented measures aimed at preventing and controlling COVID-19 ^2^. In this context, health system resources were dedicated to case management; therefore, outpatient consultations in hospitals and health facilities were suspended, including MH services ^3^.

However, in this scenario, the prevalence of depression and anxiety in Lima increased significantly, from 5.9% to 15.4% and from 3.9% to 13.3%, respectively ^4^. During the pandemic, Community Mental Health Centers (CMHC) were the main providers of MH care ^5^^,^^6^. To fulfill this function, they adapted their care services based on national regulations, which included protection measures and adapting to the use of technology for providing health services ^2^. Thus, the number of telemedicine services in the CMHC of Peru increased from 225 between January and February 2020 (pre-pandemic) to 90,000 between May and August 2020 (during the pandemic) ^7^.

Beyond this increase, it is not currently known how this care was provided and the impact it had on users and their families. In this regard, studies carried out during the pandemic in Brazil ^8^, Ecuador ^9^ and Cuba ^10^ suggest that it is essential to collect the users’ viewpoint, particularly their perceptions of the care received and their expectations and recommendations, in order to improve mental health services. Therefore, we designed a qualitative study with the aim of understanding the experiences of new and continuing users of the CMHCs of Lima and Callao, and their families, in relation to the mental health care they received during the COVID-19 pandemic.

**KEY MESSAGES**

Motivation for the study. During the pandemic, mental health consultations increased, and health systems faced new challenges in delivering care. In this context, it is important to understand how users of mental health services perceived care.

Main findings. During the pandemic, mental health care experienced changes regarding its types, resources, frequency and time, but it also faced limitations as well as benefits due to the use of technology. Users and their families saw their mental health affected.

Implications. Telephone calls to schedule and remind appointments, as well as to monitor symptoms and treatments should be maintained or implemented in Community Mental Health Centers.

## MATERIALS AND METHODS

### Design

A qualitative phenomenological study ^11^ was conducted based on an ontological paradigm, which allows researchers to describe and understand the existential dimension of the phenomena (experiences) with which the subjects coexist in their daily lives ^12^. This study was conceived, planned and executed by a team of professionals in psychology and researchers in public health and mental health, who had no relationship with the informants.

### Scope and participants

This study was part of the RESPONSE Project executed by the Center of Excellence in Chronic Diseases (CRONICAS), its aim was to understand how care was provided in four CMHCs in Lima and Callao during the pandemic. We followed a mixed methods approach, for the qualitative component we included participants of different profiles, such as: national and local decision makers, health workers, administrative workers, users and their families ^13^.

The study was carried out in four CMHCs, three in the city of Lima (North, Center and South), and one CMHC in Callao, a province adjacent to Lima. Both cities concentrate 29.9% of the Peruvian population ^14^^)^ and 47 (19%) of the 248 CMHCs existing at the national level ^5^. The CMHCs provide specialized care to people with mental disorders (common and severe) and/or psychosocial problems ^6^. When the data were collected, between September 2021 and March 2022, Peru was going through the second wave (November 2020 - October 2021) and the third wave (October 2021 - April 2022) of COVID-19, which caused a large number of infections and deaths ^15^.

We included 24 participants, users of the four CMHCs, who were selected by purposive and stratified sampling ^16^. The participants were divided into four profiles: i) new users, ii) continuing users, iii) family members of new users and iv) family members of continuing users, which were distributed as detailed in Table 1. Eight continuing users (CU) were included with the following characteristics: diagnosis of a common (e.g., anxiety, depression) or severe mental disorder (e.g., acute depression, bipolar disorder, schizophrenia), of legal age and with at least three visits in the three months prior to the declaration of the state of health emergency in Peru. In addition, we also included four family members of continuing users (FCU), of legal age and with knowledge of the care and treatment that their family member was receiving since their admission to the CMHC.

**Table 1:** Distribution of study participants.

CMHC	CMHC users		Family member of the CMHC user		Total by CMHC
	New	Continuing	New	Continuing	
Northern CMHC	2	2	1	1	6
Central CMHC	2	2	1	1	6
Southern CMHC	2	2	1	1	6
Callao CMHC	2	2	1	1	6
Total by profile	8	8	4	4	24

CMHC: Community Mental Health Center

Likewise, we included eight new users (NU) with a common or severe diagnosis, of legal age and with at least three visits after March 15, 2020 to the CMHCs, as well as four family members of these new users (FNU), of legal age and with knowledge of the care received since their admission to the CMHC.

### Techniques and instruments

Data was collected by using semi-structured interviews. The research team constructed and validated (by expert judgment) four interview guides, one for each profile (Supplementary Material). These interview guides collected information on three important topics: the impact of the pandemic on mental health, the experience of care received in CMHCs during the pandemic, and the barriers and facilitators of care, all of which were designed to understand the relationship between participants and MH services in the context of the pandemic, within the community health system. In addition, a sociodemographic data sheet was applied to each participant (age, sex, diagnosis and time of care in the CMHC).

### Procedures

Authorization was requested from the Health Directorates in charge of the CMHCs and from the head of the center; then health workers helped to identify and invite users and family members who met the inclusion criteria. Those who showed interest were contacted by telephone to provide informed consent by email or WhatsApp, and received information regarding the study objectives and procedures. Those who agreed to participate provided their consent orally, which was recorded and stored, then the interviews were scheduled. Those who were unable to provide consent were excluded.

The interviews were conducted between September 2021 and March 2022 by three researchers with experience in qualitative research and mental health. All interviews were conducted in a single session via phone call or Zoom video call, they averaged 32 minutes in length and were audio recorded.

### Data analysis

Once transcribed, we carried out a thematic analysis of the interviews ^17^. Initially, two team members (NC and AV) did deductive coding ^18^ of four interviews (one per profile), using the interview guide as a basis to create a first codebook. Subsequently, four team members (NC, AV, VC and RP) developed the second version of the codebook. This was used by two researchers (NC and AV) to do an inductive coding of four interviews, using the ATLAS.ti version 9 program ^19^, in which they achieved more than 95% agreement in the concordance coefficient during the coding process of identical units of analysis, which allowed identifying the triangulation of information between CMHC users and their relatives. Finally, each of the researchers (NC, AV) analyzed the interviews individually, achieving data saturation in all categories of analysis.

The information was organized into the categories and themes detailed in Table 2.

**Table 2:** Categories and topics of analysis according to participant profile.

Categories	Topics	Continuing users and family members	New users and family members
Impact of the pandemic on MH	Impact of sanitary measures	X	X
	Impact of the suspension of face-to-face care at CMHCs	X	-
CMHC care	Return of MH care services	X	-
	Reason for consultation and route of access to CMHC		X
	Use of technology in CMHC pre-pandemic services	X	-
	Use of technology in CMHC services during the pandemic	X	X
	Services provided by CMHC during the pandemic	X	X
	Characteristics of healthcare prior to the pandemic	X	-
	Characteristics of healthcare during the pandemic	X	X
Limitations in care during the pandemic	Difficulties in the use of technology	X	X
	Limitations in the quality of care	X	X
	High demand for care	X	X
Facilitators in Pandemic Care	Work carried out by CMHC employees	X	X
	Use of technology in care	X	X
Recommendations for improving mental health care in CMHC	Suggestions from users and family members to improve care at CMHCs	X	X

CMHC: Community mental health center; MH: Mental health.

### Ethical considerations

The study was approved by the Institutional Ethics Committee of the Universidad Peruana Cayetano Heredia, Lima (Record of approval No. 573-33-20). All participants provided informed consent prior to the interview. The team completed a course on ethics and good clinical practices.

## RESULTS

### Impact of the pandemic on users of CMHCs

COVID-19 was associated with “death”, “grief”, “contagion” and “confinement” among new and continuing users due to the context caused by the virus in the country (high mortality rates and high number of cases; this being the main cause of emotional impact (sadness, anguish, fear) on users. Likewise, the emotional impact was also associated with concern about the continuity of their care among continuing users, mainly due to not knowing “when” and “how” their treatment would be resumed on a regular basis.

*At the beginning, when the pandemic started, well, everything was up in the air, that is, I, the truth is that I ran out of pills at the beginning, without appointments. [... ] they closed down completely, I was a little worried about the pills* (CU 02, CMHC Northern Lima).

The majority of users (new and continuing) perceived that the pandemic and the need to adapt to health measures exacerbated pre-existing symptoms in people with mental health problems, a perception that was ratified by their family members, who accompanied users as their primary caregivers during the pandemic.

In this regard, family members of users (new and continuing) mentioned that this accompaniment meant an increase in the burden of their activities, which were related to the supervision of compliance and adherence to treatment (mainly pharmacological and other types of care during the pandemic), compliance with health regulations (use of biosecurity implements, social isolation, curfews, among others); and the accompaniment and socio-affective support to users. This caused a negative impact on the mental health of family members.

*(...) And we had to be locked up, could not to go out during quarantine. It was very chaotic, the confinement, and that also depresses, there are changes in people, even if they do not have a psychological problem. We have all been affected in one way or another, and rightly so for the patient* (FCU 02, CMHC Callao).

### Care at CMHCs during the pandemic

All continuing users and their families mentioned that during the pandemic, face-to-face activities at the CMHCs were suspended, these activities included requesting information, scheduling appointments, individual and group care, and dispensing medications. After the declaration of the state of emergency, users of the CMHCs in North, South and Central Lima reported that health personnel telephoned to inform them that, from now on, their care would be virtual. However, a continuing user of CMHC in Northern Lima, with whom they did not communicate by telephone, learned through an acquaintance that the CMHC had been reopened, exclusively for the delivery of medicines. Finally, users of the Callao CMHC attended in person to request information, since they were not contacted by their CMHC.

*As of March, there were no appointments at all. The following month, they just implemented the phone calls, and that, and that, I think it took a while more (...) for the phone appointments to come out* (CU 01, CMHC Southern Lima).

Five of the eight new users had no previous history of mental health care, although most of them had previously experienced symptoms that were exacerbated during the pandemic. For that reason, after learning, through friends, family or social networks, about the services offered at the CMHCs, they decided to seek help. Other two users were referred to a CMHC because their usual health facilities interrupted mental health care. Finally, one woman was referred from the prosecutor’s office due to domestic violence.

*I didn’t know that I was mentally ill. When a psychologist friend came to visit me, I told him ‘I am married’, but I am single, so I had hallucinations [... ] he told me: ‘It seems to me that you have short-circuited your brain’ [... ] and that it would be good for you to be taken to a psychiatrist. Then he gave me the address of the CMHC. I went and they treated me without any problems* (NU 01, CMHC Southern Lima).

Regarding the scheduling of appointments, continuing users report that, prior to the pandemic, this process was done in person. However, all informants agreed that, throughout the pandemic, appointments were scheduled by phone call, during which the patient verified the availability of appointments, dates, times and type of care.

*They had called me once, twice... that one day there was not going to be any care because the doctor did not come...or also reminding me that I had to go the next day... or since it is at night, I forgot and they reminded me that the next day at a certain time I had an appointment* (NU 01, CMHC Northern Lima).

Users agreed that CMHC staff monitored, above all, their emotional state and compliance with pharmacological treatment during the pandemic. The majority of users and family members identified that the offer of face-to-face or virtual care in the CMHCs depended on the pandemic situation (waves of contagion), which influenced, in addition to the type of care, its frequency, the prescription of certain medications and the scarcity of others, as well as the referral to specific services such as psychology, psychiatry or medicine, together with the perception of an increase in the demand for care from the CMHC services.

*They called me more often to ask how I was doing, how I was, my emotions, in the case of the psychologist. The psychiatrist only every time they had to medicate me [... ] The truth is that in pandemic times they have been more attentive to everyone* (CU 01, CMHC Central Lima).

*They (CMHC workers) are giving us the medicine for the user, but since there is no Olanzapine, I have to buy it myself, because there is none, we are going to Noguchi (Hospital) to buy it there because it is more convenient* (FCU, CMHC Northern Lima).

In this regard, continuing users receiving pharmacological treatment reported that, at the beginning of the pandemic (March - May 2020), they first contacted their CMHCs by telephone and, although initially they were not offered to resume their individual care, they were able to schedule the pick-up of their medication, and in some cases, they extended the validity of their prescriptions to reduce their attendance at the CMHC and avoid exposing them to possible contagion. In addition, some users were able to pick up their medications at the CMHCs after receiving a photo, via WhatsApp, of their physical prescriptions signed by the treating physician.

*The doctor would tell me ‘I am going to send you your medication for 2 months because you can’t keep coming back’ Then, he would tell me ‘You are going to pick up your prescription at the health center’. I went with my ID card, they gave me the prescription and gave me the medicine with the indications* (CU 01, CMHC Callao).

Subsequently, as pointed out by new and continuing users, CMHCs prioritized individual attention through video calls via Zoom, Meet or WhatsApp. However, this method presented some difficulties described in the following section, so it was replaced by phone calls. A few months later, between the first wave and the first months of the second wave (August - December 2020), some CMHCs in South Lima, Downtown and Callao resumed face-to-face psychological and psychiatric care. Users in Callao mentioned that their visits lasted up to 45 minutes, while users in Southern Lima, Central Lima and Northern Lima mentioned that their visits lasted between 15 and 30 minutes. Users agreed that, on average, the frequency of service was every three weeks. However, during the peak of the second wave (December 2020 - April 2021), and during the first months of the third wave (January - March 2022), remote attention was prioritized again, mainly via phone calls, which lasted an average of 15 minutes every two weeks on average.

*We would make telephone appointments... and he would call me (CMHCs worker), it was once a month that they met with me (in person), they would ask me how I was doing. And then he would say to me: Are you going to take the same medication? And with this pill, he would tell me, at any time I had to go to the center to pick up the prescription, nothing else (CU 01, CMHC Southern Lima)*.

Overall, most continuing users did not perceive important differences in the duration and frequency of their care compared to before the pandemic, and perceived that the use of phone calls and WhatsApp messaging was transversal to the types of care they received during the pandemic.

### Difficulties in Care During the Pandemic

The majority of participants considered that the main difficulty during the pandemic was adapting to video calls to resume their individual care at the CMHCs. This may be due to the following reasons described by users: the low quality of the Internet connection in their homes, the expense of recharging Internet data to access video calls, and their unfamiliarity with the use of platforms such as Zoom or Meet. For these reasons, users identified that video calls, as a type for individual attention, lasted only a short time.

*As I was sharing data, for example, my Internet sometimes did not work well... I had to make an additional cost to put “Internet Hogar” (a different internet service), with more megabytes, to be able to access these sessions* (CU 01, CMHC Central Lima).

*If we could not make video calls, they opted for phone calls, both the psychologist and the psychiatrist* (CU 01, CMHC Callao*)*.

Another difficulty perceived by both groups of users was related to the quality of virtual services. Emphasis was placed on their preference for face-to-face care, since this type of care meant a more direct contact with their providers; and accessing the CMHC facilities to receive their consultations was related to the possibility of having a private and intimate space. In this sense, given that virtual care did not have these characteristics, it was perceived as a having less effectiveness for providing treatment.

*What I do [... ] a little bit is my treatment, because when it was face-to-face it was better...I felt that the treatment was working better, in a better way... That it progressed in a better way. On the other hand, virtual, no...no, I didn’t think so* (CU 01, CMHC Central Lima).

Another difficulty reported by users was the increase in the demand for care, which was described as the main cause of the saturation of the CMHC telephone lines. This meant that appointments scheduled by telephone took a long time to be answered by the CMHC staff, or else they ran out quickly, requiring extra appointments or, in some cases, reducing the frequency of service.

*There were many patients who were calling ... it took up the whole morning. There were days when I would call all morning and they would answer only in the afternoon, and it was a bit tedious* (CU 01, CMHC Southern Lima).

### Facilitators and benefits of care during the pandemic

All informants emphasized that the work performed by CMHC staff enabled compliance and continuity of their treatment. They agreed that, despite the fact that the staff had to face their own challenges and expose their lives to continue care, they did not “give up” in their work. In addition, they reported that, at all times, the CMHC staff sought alternatives so that the users could be attended, providing them with extra appointments and/or calling them constantly.

*You feel important, that they care about you, don’t you? At no time, as I tell you, they did not faint, they did not take the pandemic as a pretext to leave the patient, not at all. On the contrary, they were there more alert than ever* (CU 01, CMHC Central Lima).

The majority of participants highlighted that the use of phone calls to monitor them, as well as to schedule and remember appointments, was beneficial and helped users feel “more accompanied”. Finally, although the lines were frequently overcrowded, they felt that scheduling their appointments by phone was advantageous because they avoided lines and crowds inside CMHC.

*They (CMHC workers) cared about my health and made me feel that I was important* (CU 01, CMHC Callao).

*But they have always been present (CMHC workers) (...) I always liked to see them, because, this, I don’t know, I was, I needed to see them in order to feel good, they always called me to schedule the appointment. And they always called me to remind me* (CU 01, CMHC Central Lima).

### Recommendations for care at the CMHC

When asked about their recommendations for improving care at the CMHCs, users and their families considered it important to reopen group activities, such as the “psychosocial club”, a space where users are welcomed and accompanied in their process of integration into the community. At the time of the interviews, these activities had been resumed, albeit virtually, only in one CMHC. In addition, several users, while virtual services were predominant, recommended that face-to-face services be resumed. A third recommendation was that telephone calls should continue, mainly to monitor their mental health status, remind them of appointments and coordinate the delivery of medications. Finally, some users and family members recommended establishing communication channels between users and CMHC through social networks.

## DISCUSSION

Our study describes the experiences of CMHC users and their family members regarding mental health care provided at CMHCs during the pandemic.

The participants perceived a worsening of emotional discomfort among users, linked to limitations in their daily activities and uncertainty about the continuity of their care, but also to the fear of catching COVID-19, or losing a family member to the disease. The framework of these experiences are the strict restrictions that were implemented in Peru, such as the curfews ^20^, but also the high COVID-19 mortality rate, which was one of the highest in the world for a long time ^15^. In line with our results, a study in China, with non-psychiatric and psychiatric patients, concluded that the latter presented higher symptoms of stress, depression, anxiety and insomnia during the pandemic, and more concerns about their physical health, irritability and suicidal ideation during the months with more restrictions ^21^.

Moreover, in this context, family members of users experienced an increase in their workload and emotional exhaustion, which coincides with a study conducted in Mexico, which showed that the burden of caregivers of people with disabilities, especially those with chronic diseases, increased during the pandemic ^22^.

An important result is the identification of changes in the care offered by CMHCs during the pandemic. In Peru, face-to-face mental health care was interrupted due to the COVID-19 pandemic, as was the case in most countries of the world ^23^. Thus, although Peruvian regulations prioritized mental health care, they did not contemplate such care in the midst of a health emergency, making it necessary to create guidelines to guarantee this care using technology ^6^. Something similar was evident in 70% of the countries of the world, which adopted telemedicine as the main way to provide mental health care during the pandemic ^24^.

However, the Peruvian health system does not have adequate equipment and technology, so many users lack the equipment, services and resources to access telemedicine, which limited its use mainly to telephone calls and messages from the CMHCs to schedule individual virtual or face-to-face care, appointment reminders and to monitor the health status of users. Even so, telephone calls were a feasible and effective strategy; moreover, they were well received by users in Peru. A similar context was reported in Mexico, where video calls were also sometimes replaced by telephone calls, which were more accessible to users ^24^. Phone calls were also used in wealthy countries, such as Australia, where they were used for remote consultations in nursing, psychiatry, psychology and social work, with good user acceptance ^25^.

Our findings reveal a preference for face-to-face care over virtual care. International evidence on the acceptance of telemedicine services is heterogeneous. On the one hand, it is considered that it may affect the therapeutic link between user and provider, and hinder the evaluation of certain signs and symptoms, while, on the other hand, it offers continuity of care in mental health ^26^. Among CMHC users, the lower preference for virtual care could be due, in part, to the difficulties they faced in having an adequate Internet connection during remote consultations.

It is important to highlight that implementing technological strategies to the health system requires additional investment, although according to the World Health Organization, only 17% of the countries had extra funding for the requirements for the implementation of telemedicine during the pandemic ^23^. In Peru, although there was a larger budget allocated for mental health services, the available information does not reveal whether this funding was used to provide CMHCs with equipment, Internet connection and to train personnel, which are strategies recommended by international guidelines ^26^.

The use of technology during the pandemic allowed for continuity of treatment for CMHC users, but faced several limitations. Since mental health is a state priority, but also an essential aspect during emergencies such as the pandemic, the health system must invest in equipment and telephone and Internet services, as well as in training, to ensure that providers, users and family members can offer and receive remote care without difficulties. The evidence reveals that, although telemedicine requires a restructuring of the system and a financial investment to start, telemedicine services in mental health prove to be cost-effective in the medium term ^27^.

Furthermore, given that the impact of using telephone calls to schedule and remember appointments, as well as to monitor symptoms and treatments was positive and has shown good results in other countries ^25^, it should be maintained and improved in CMHCs. One could, for example, also use calls to monitor emotional state, with standardized questions, as other studies recommend ^28^.

Likewise, taking into account the overload that the pandemic placed on caregivers, it is important to offer them both guidance to accompany users and emotional support to protect them and allow them to continue with their caregiving work ^29^.

One of the limitations of the study is that the research group had to virtually guide users to solve problems regarding the use of the Zoom platform, and in some cases, video calls had to be adapted to telephone calls, due to the resources of the users. The interviews were conducted at different times, during the second and third waves of the pandemic, so participants’ experiences could differ, in part, due to each different context. Recall bias may have also had an effect on our results due to the time elapsed since the onset of the pandemic. However, the interviews were conducted with a semi-structured form that allowed guiding the participant through the different moments of their mental health care. A strength of the study was the participation of family members of the users (new and continuing), which allowed the information to have greater support.

In conclusion, this study, which collected narratives from different stakeholders (new patients, continuing patients and their families), found that the COVID-19 pandemic, health and social restrictions, and the closure of mental health facilities exacerbated the symptoms of people with mental health problems and increased the emotional, physical and social burden on caregivers. After face-to-face appointments were suspended, care at CMHCs was resumed with the support of technology, mainly telephone calls and mobile messaging, which were used to provide individual care, schedule and remind appointments, and monitor the emotional state and treatment of users. The limitations described by users regarding their care were: increased demand for care, difficulties in accessing video calls, and a perception that virtual care had less impact on their recovery. However, we also identified facilitators for care, such as the commitment of health personnel and the frequent use of telephone calls, which allowed users to feel accompanied during the pandemic context.
