# Supplementary material for: Experiences of users and family members on the care received at community mental health centers in Lima and Callao during the COVID-19 pandemic
Source: Rev Peru Med Exp Salud Publica. 2023 Sep 25;40(3):278–86. doi: 10.17843/rpmesp.2023.403.12717 (PMC10953668; doi:10.17843/rpmesp.2023.403.12717)
Supplement: Supplementary material. — Available in the electronic version of the RPMESP. [file rpmesp-40-03-12717-s001.docx]

**Material suplementario: Guías de entrevistas diferenciados por perfiles.**

**Guía de entrevista a Usuarios Nuevos**

**Experiencia sobre la atención recibida en el CSMC**

Ahora me gustaría hacerle algunas preguntas sobre su experiencia en el CSMC.

1. **¿Podría comentarme cómo llegó al CSMC, cómo fue su primera cita?** *Explorar cómo se enteró del CSMC y si esta búsqueda se vio influenciada por otros (familiares, amigos, noticias, redes sociales, derivación de otro centro de salud), explorar qué tan sencillo, rápido, eficiente fue el iniciar su atención, explorar el motivo de consulta*

***Si aún no lo ha dicho***

1. *¿Cómo es que decide buscar atención en este CSMC? Explorar si tenía ideas previas, se lo habían recomendado, fue su último recurso, etc.*
2. *Los diversos momentos de la pandemia, ¿afectaron de alguna manera el momento en que decidió o pudo buscar atención aquí? Por ejemplo, si debido a la cuarentena postergó la búsqueda de atención o no logró conseguir una consulta o fue atendida por otra ruta*
3. **¿Podría contarme ahora cómo ha sido la atención que ha ido recibiendo? Con atención nos referimos a citas con algún trabajador de salud, llamadas telefónicas, consultas virtuales, actividades grupales, visitas a su casa, recojo de medicamentos, etc.** *Explorar qué servicios ha recibido, con cuántos trabajadores de salud se ha atendido, cómo son esas atenciones (virtuales, presenciales, telefónicas), cómo es el recojo de medicinas (si las tomara), visitas domiciliarias, actividades grupales. Explorar si usó otros medios virtuales (SMS, redes sociales, zoom, etc.) Conocer si el CSMC se ha comunicado con algún familiar suyo*
4. **Desde la experiencia que me viene relatando ¿Cómo le han parecido las atenciones? ¿Recibió la atención que esperaba, se siente satisfecho/a? ¿Le gustaría que estos cambios se mantuvieran aún después de la pandemia?** *Explorar aspectos positivos y negativos de la atención recibida, incluyendo la calidad, si cree que le ayuda, percepción de mejora sobre el motivo de consulta inicial u otros posibles temas que hayan surgido en el tiempo*
5. **Algunos servicios deben haber cambiado a partir de la pandemia, ¿usted notó o se vio afectado por alguno de estos cambios?** *Por ejemplo, tal vez no conseguía citas porque había menos trabajadores; o por el contrario, tal vez pudo ahorrar tiempo al tener las consultas virtuales y no tener que movilizarse. O* si cambiaron los trabajadores que lo fueron atendiendo, o tal vez tuvo más dificultades durante los primeros meses de la pandemia

**Barreras y facilitadores para la atención y servicios brindados**

Ahora me gustaría hablar un poco sobre los problemas o dificultades que tuvo en su atención y también sobre aquellas cosas que ayudaron.

1. **¿Qué dificultades encontró para su atención en el CSMC?** *Explorar sobre acceso a los servicios y medicamentos, consultas virtuales, llamadas telefónicas, gestionar una cita, contactar a un trabajador de salud.*
2. *¿Siente que estos problemas mejoraron con el tiempo?*
3. **¿Qué cosas cree que ayudaron a que el CSMC siga atendiendo?** *Explorar sobre organización de los trabajadores, iniciativas de los trabajadores y/o usuarios*
4. **¿Qué recomendaciones tendría para mejorar la atención del CSMC? ¿Le gustaría agregar algo?**

**Guía de entrevista a Usuarios Continuadores.**

**Descripción y cambios en la atención recibida en el csmc**

Me gustaría hacerle algunas preguntas sobre la atención que ha ido recibiendo en el CSMC. Para eso, me gustaría que podamos diferenciar antes y durante la pandemia por COVID-19:

- ANTES del inicio de la pandemia, es decir antes de marzo 2020;
- DURANTE la pandemia, es desde marzo de 2020 en adelante. Al ser un periodo de tiempo amplio y debido a que las medidas para hacer frente a la pandemia han sido diferentes, quisiéramos explorar las diferencias que haya habido a lo largo del tiempo. Por ejemplo, hacer la distinción de tres momentos: durante la cuarentena (marzo - junio 2020) el resto del 2020, y durante la segunda ola (febrero-mayo 2021) en adelante.

1. **¿Podría contarme cómo era la atención en el CSMC ANTES de la pandemia, es decir, antes del 15 de marzo del 2020?** **Con atención nos referimos a citas con algún trabajador de salud, llamadas telefónicas, consultas virtuales, actividades grupales, visitas a su casa, recojo de medicamentos, procedimientos para agendar una cita, etc.** *Explorar con qué trabajadores de salud se atendía, qué atenciones recibía, con qué frecuencia se atendía*
2. **De todo lo que me ha contado, ¿qué cosas cambiaron a partir de la pandemia, es decir, desde marzo 2020 en adelante?** *Explorar si siguió qué servicios ha recibido o cuales ha dejado de recibir, con cuántos trabajadores de salud se ha atendido, como son esas atenciones (virtuales, presenciales, telefónicas), cómo es el recojo de medicinas (si las tomara), visitas domiciliarias y actividades grupales. Si los trabajadores se han comunicado con algún familiar suyo.*
3. **¿Qué le parecieron estos cambios en las atenciones?** **En general, ¿considera que ha recibido la atención que esperaba, se siente satisfecho/a? ¿Le gustaría que estos cambios se mantuvieran aún después de la pandemia?** *Explorar aspectos positivos y negativos de la atención recibida, incluyendo la calidad, si cree que le ayuda, percepción de mejora sobre el motivo de consulta inicial u otros posibles temas que hayan surgido en el tiempo*

***Explorar aspectos positivos y negativos de estos cambios***

1. ***¿Considera que estos cambios afectaron su estado de salud mental o el avance de su tratamiento?*** *Por ejemplo, ¿sintió retrocesos o más dificultades que antes; o por el contrario, tal vez más acompañamiento o cosas que funcionaron mejor?*

**Barreras y facilitadores para la atención y servicios brindados**

Ahora me gustaría hablar un poco sobre los problemas o dificultades que tuvo en su atención y también sobre aquellas cosas que ayudaron.

1. **¿Qué dificultades encontró para su atención en el CSMC?** *Explorar sobre acceso a los servicios y medicamentos, consultas virtuales, llamadas telefónicas, gestionar una cita, contactar a un trabajador de salud.*
2. *¿Siente que estos problemas mejoraron con el tiempo?*
3. **¿Qué cosas cree que ayudaron a que el CSMC siga atendiendo?** *Explorar sobre organización de los trabajadores, iniciativas de los trabajadores y/o usuarios*
4. **¿Qué recomendaciones tendría para mejorar la atención del CSMC? ¿Le gustaría agregar algo?**

**Guía de entrevista a Familiares de Usuarios Nuevos**

**Experiencia sobre la atención recibida en el CSMC**

Ahora me gustaría hacerle algunas preguntas sobre su experiencia en el CSMC.

1. **¿Podría comentarme cómo llegó su familiar al CSMC, cómo es que inicia su atención?** Explorar cómo su familiar se enteró del CSMC, qué otras opciones se consideraron y si esta búsqueda se vio influenciada por otros (familiares, amigos, noticias, redes sociales, derivación de otro centro de salud)
2. **Los diversos momentos de la pandemia, ¿afectaron de alguna manera el momento en que su familiar decidió o pudo buscar atención aquí?** *Por ejemplo, si debido a la cuarentena su familiar postergó la búsqueda de atención o no logró conseguir una consulta o fue atendida por otra ruta*
3. **¿Podría contarme cómo ha sido la atención que ha ido recibiendo su familiar en el CSMC?** *Explorar qué servicios ha recibido, con cuántos trabajadores de salud se ha atendido, como son esas atenciones (virtuales, presenciales, telefónicas), cómo es el recojo de medicinas (si las tomara), visitas domiciliarias, actividades grupales*
4. **¿Qué opina usted de la atención que se le viene brindando a su familiar en el CSMC? En general, ¿considera que ha recibido la atención que esperaba, se siente satisfecho/a? ¿Le gustaría que estos cambios se mantuvieran aún después de la pandemia?** En este tiempo, **¿los trabajadores de salud se han comunicado con usted?**
5. **Algunos servicios deben haber cambiado a partir de la pandemia**, **¿Usted notó que su familiar se vio afectado por alguno de estos cambios?** *Por ejemplo, tal vez no conseguía citas porque había menos trabajadores; o por el contrario, tal vez pudo ahorrar tiempo al tener las consultas virtuales y no tener que movilizarse. O quizá que fueron cambiando los trabajadores que fueron atendiendo a su familiar , o tal vez tuvo más dificultades durante los primeros meses de la pandemia*
6. **¿Considera que estos cambios afectaron su estado de la salud mental de su familiar?** *Por ejemplo, ¿sintió retrocesos o más dificultades que antes; o por el contrario, tal vez más acompañamiento o cosas que funcionaron mejor?*

**Barreras y facilitadores para la atención y servicios brindados**

Ahora me gustaría hablar un poco sobre los problemas o dificultades que hubo a partir de la pandemia, y también sobre aquellas cosas que ayudaron.

1. **¿Qué dificultades encontró en la atención DURANTE la pandemia? ¿Siente que estos problemas mejoraron con el tiempo?** *Explorar sobre acceso a los servicios y medicamentos, consultas virtuales, llamadas telefónicas, gestionar una cita, contactar a un trabajador de salud.*
2. **¿Qué cosas cree que ayudaron a que el CSMC siga haciendo su trabajo?** *Explorar sobre organización de los trabajadores, iniciativas de los trabajadores y/o usuarios.*

**Percepción sobre el impacto de la cuarentena en la salud mental de su familiar**

Me gustaría conversar sobre la forma en que la pandemia por el coronavirus puede haberle afectado a su familiar emocionalmente, en sus síntomas o en su tratamiento.

1. **¿Considera que la pandemia ha afectado de alguna manera la salud mental de su familiar o su estado de ánimo? Sea emocionalmente, en sus síntomas o en su tratamiento. ¿Cómo así?** *Si indica impacto, explorar cómo han sobrellevado la situación, medidas en casa o con familia para manejarlo.*
2. **¿Esta situación de pandemia le ha afectado emocionalmente también a usted? ¿Cómo así?**
3. **¿Considera que el tener que cuidar a su familiar en el contexto de la pandemia puede haber afectado también la salud mental de usted o su familia?**
4. **¿Hay algo más que le gustaría agregar?**

**Guía de entrevista a Familiares de Usuarios Continuadores**

**Descripción y cambios en la atención recibida en el csmc**

Me gustaría hacerle algunas preguntas sobre la atención que su familiar ha ido recibiendo en el CSMC. Para eso, me gustaría que podamos diferenciar antes y durante la pandemia por COVID-19:

- ANTES del inicio de la pandemia, es decir antes de marzo 2020;
- DURANTE la pandemia, es desde marzo de 2020 en adelante. Al ser un periodo de tiempo amplio y debido a que las medidas para hacer frente a la pandemia han sido diferentes, quisiéramos explorar las diferencias que haya habido a lo largo del tiempo. Por ejemplo, hacer la distinción de tres momentos: durante la cuarentena (marzo - junio 2020) el resto del 2020, y durante la segunda ola (febrero-mayo 2021) en adelante.

1. **¿Podría contarme cómo era la atención en el CSMC ANTES de la pandemia, es decir, antes del 16 de marzo del 2020**? *Explorar con qué trabajadores de salud se atendía su familiar, qué atenciones recibía, con qué frecuencia se atendía*
2. **De todo lo que me ha contado, ¿qué cosas cambiaron a partir de la pandemia, es decir, desde marzo 2020 en adelante?** *Explorar qué servicios ha recibido, con cuántos trabajadores de salud se ha atendido, como son esas atenciones (virtuales, presenciales, telefónicas), cómo es el recojo de medicinas (si las tomara), visitas domiciliarias, actividades grupales.*
3. **¿Qué le parecieron estos cambios en las atenciones? En general, ¿considera que ha recibido la atención que esperaba, se siente satisfecho/a? ¿Le gustaría que estos cambios se mantuvieran aún después de la pandemia?** *Explorar aspectos positivos y negativos de estos cambios o si sintió que los cambios afectaron a su familiar*
4. A partir de algunos cambios en los servicios del CSMC debido a la pandemia, **¿Usted notó que su familiar se vio afectado por alguno de estos cambios?** *Por ejemplo, tal vez no conseguía citas por menos trabajadores; o por el contrario, tal vez pudo ahorrar tiempo al tener las consultas virtuales y no tener que movilizarse. O quizá que fueron cambiando los trabajadores que fueron atendiendo a su familiar , o tal vez tuvo más dificultades durante los primeros meses de la pandemia*
5. **En este tiempo, ¿los trabajadores de salud se han comunicado con usted? ¿De qué hablaban? ¿Cuán frecuente era esto?**

**Barreras y facilitadores para la atención y servicios brindados**

Ahora me gustaría hablar un poco sobre los problemas o dificultades que hubo a partir de la pandemia, y también sobre aquellas cosas que ayudaron.

1. **¿Qué dificultades encontró en la atención DURANTE la pandemia? ¿Siente que estos problemas mejoraron con el tiempo?** *Explorar sobre acceso a los servicios y medicamentos, consultas virtuales, llamadas telefónicas, gestionar una cita, contactar a un trabajador de salud.*
2. **¿Qué cosas cree que ayudaron a que el CSMC siga haciendo su trabajo?** *Explorar sobre organización de los trabajadores, iniciativas de los trabajadores y/o usuarios.*

**Percepción sobre el impacto de la cuarentena en la salud mental de su familiar**

Me gustaría conversar sobre la forma en que la pandemia por el coronavirus puede haberle afectado a su familiar emocionalmente, en sus síntomas o en su tratamiento.

1. **¿Considera que la pandemia ha afectado de alguna manera la salud mental de su familiar o su estado de ánimo? Sea emocionalmente, en sus síntomas o en su tratamiento. ¿Cómo así?** *Si indica impacto, explorar cómo han sobrellevado la situación, medidas en casa o con familia para manejarlo.*
2. **¿Esta situación de pandemia le ha afectado emocionalmente también a usted? ¿Cómo así?**
3. **¿Considera que el tener que cuidar a su familiar en el contexto de la pandemia puede haber afectado también la salud mental de usted o su familia?**
4. **¿Hay algo más que le gustaría agregar?**
