# Supplementary material for: Experiences of users and family members on the care received at community mental health centers in Lima and Callao during the COVID-19 pandemic
Source: Rev Peru Med Exp Salud Publica. 2023 Sep 25;40(3):278–86. doi: 10.17843/rpmesp.2023.403.12717 (PMC10953668; doi:10.17843/rpmesp.2023.403.12717)
Supplement: Translation

# Translation: Experiencias de usuarios y familiares sobre la atención recibida en centros de salud mental comunitaria de Lima y Callao durante la pandemia de la COVID-19

**Authors:** Noelia Cusihuaman-Lope, Ana L. Vilela-Estrada, Victoria Cavero, David Villarreal-Zegarra, Francisco Diez-Canseco

**Affiliations:** 1 CRONICAS Centro de Excelencia en Enfermedades Crónicas, Universidad Peruana Cayetano Heredia, Lima, Perú.; 2 Instituto Peruano de Orientación Psicológica, Lima, Perú.

**Keywords:** Atención, Salud Mental, Usuarios, Cuidadores, Servicios de Salud Comunitaria

## Abstract

**Objetivos.:**

Comprender las experiencias de usuarios nuevos y continuadores de los Centros de Salud Mental Comunitaria (CSMC) de Lima y Callao, y de sus familiares, en relación a la atención en salud mental que recibieron durante la pandemia de la COVID-19.

**Materiales y métodos.:**

Estudio cualitativo realizado entre septiembre del 2021 y febrero del 2022, en el que se entrevistó a 24 usuarios y familiares que interactuaron con los servicios brindados por tres CSMC de Lima y uno del Callao, durante la pandemia de la COVID-19. Se realizó un análisis temático de las entrevistas transcritas.

**Resultados.:**

Los informantes percibieron que la pandemia exacerbó los síntomas de las personas con problemas de salud mental. Durante la pandemia, las atenciones de salud mental se apoyaron en el uso de tecnología, principalmente de llamadas telefónicas, las que sirvieron para monitorear el estado emocional y el tratamiento farmacológico de los usuarios, así como para programar y recordar citas. Los usuarios destacan que las llamadas telefónicas frecuentes les hicieron sentirse acompañados y resaltan el compromiso de los trabajadores de los CSMC. Como dificultades, reportan el incremento en la demanda de atención, problemas para acceder a videollamadas, y menor calidad en las atenciones virtuales.

**Conclusiones.:**

La COVID-19 impactó emocionalmente a las personas con problemas de salud mental, a su vez, los servicios de los CSMC vieron afectada la modalidad (presencial o virtual), recursos, frecuencia, tiempo y calidad de la atención, encontrando limitaciones y beneficios en el uso de la tecnología.

## INTRODUCCIÓN

Antes de la pandemia de la COVID-19, el 20% de la población peruana presentaba algún trastorno mental, y solo 3 de cada 10 residentes de Lima y del Callao que requerían atención en salud mental (SM) la recibían ^1^. El 15 de marzo del 2020, el Gobierno peruano declaró el Estado de Emergencia Nacional e implementó medidas destinadas a prevenir y controlar la COVID-19 ^2^. En ese contexto, los recursos del sistema de salud se enfocaron en la atención de casos, generando como medida preliminar la suspensión de las consultas ambulatorias presenciales en hospitales y establecimientos de salud, incluyendo los servicios de SM ^3^.

Sin embargo, durante este escenario la prevalencia de depresión y ansiedad en Lima se incrementó significativamente, pasando, de 5,9% a 15,4% y de 3,9% a 13,3%, respectivamente ^4^. Durante la pandemia, los Centros de Salud Mental Comunitaria (CSMC), fueron los principales encargados de brindar atención en SM ^5^^,^^6^. Para cumplir dicha función adaptaron sus servicios de atención basados en las normativas nacionales; que incluían medidas de protección y la adaptación al uso de tecnología como la principal herramienta para brindar servicios de salud ^2^. Así, el número de atenciones de telemedicina en los CSMC del Perú, pasó de 225 entre enero y febrero del 2020 (prepandemia), a 90 000 entre mayo y agosto del 2020 (durante la pandemia) ^7^.

Más allá de este incremento, en la actualidad no se conoce cómo se efectuaron estas atenciones y el impacto que tuvo en los usuarios y sus familiares. Al respecto, estudios realizados durante el contexto de pandemia en Brasil ^8^, Ecuador ^9^ y Cuba ^10^ sugieren que es esencial recoger la mirada de los usuarios, en particular sus percepciones sobre la atención recibida y sus expectativas y recomendaciones, para mejorar los servicios de salud mental. Guiados por ese interés, diseñamos un estudio cualitativo con el objetivo de comprender las experiencias de usuarios nuevos y continuadores de los CSMC de Lima y Callao, y de sus familiares, en relación a la atención en salud mental que recibieron durante la pandemia de la COVID-19.

**MENSAJE CLAVE**

Motivación para realizar el estudio. Durante la pandemia, las atenciones en salud mental se incrementaron, y los sistemas de salud presentaron nuevos retos para lograr sus atenciones. En este contexto, resulta importante comprender cómo percibieron sus atenciones los usuarios de servicios de salud mental.

Principales hallazgos. Durante la pandemia, las atenciones en salud mental cambiaron de modalidad, recursos, frecuencia y tiempo, además, las atenciones también presenciaron limitaciones y a su vez beneficios en el uso de la tecnología. Los usuarios y sus familiares vieron afectada su salud mental.

Implicancias. Las llamadas telefónicas para programar y recordar citas, así como monitorear síntomas y tratamientos debería mantenerse o instaurarse en los Centros de Salud Mental Comunitaria.

## MATERIALES Y MÉTODOS

### Diseño

Se realizó un estudio cualitativo fenomenológico ^11^ basado en un paradigma ontológico; lo cual permite a los investigadores describir y comprender la dimensión existencial de los fenómenos (experiencias) con los cuales los sujetos conviven en su cotidiano ^12^. El estudio fue ideado, planificado y ejecutado por un equipo de profesionales en psicología e investigadores en salud pública y salud mental, quienes no tuvieron ninguna relación con los informantes.

### Ámbito y participantes

El presente estudio fue parte del Proyecto RESPONSE ejecutado por el Centro de Excelencia en Enfermedades Crónicas (CRONICAS); para entender cómo fue la atención en cuatro CSMC de Lima y Callao, durante la pandemia. El proyectó fue trabajado mediante una metodología mixta, y para el componente cualitativo se incluyeron a participantes de diversos perfiles como: tomadores de decisiones nacionales y locales, trabajadores de salud, trabajadores administrativos, usuarios y sus familiares ^13^.

El estudio se realizó en cuatro CSMC que incluyeron a tres en la ciudad de Lima (Norte, Centro y Sur), y un CSMC del Callao, provincia colindante con Lima. Ambas ciudades concentran al 29,9% de la población peruana ^14^ y a 47 (19%) de los 248 CSMC existentes a nivel nacional ^5^. Los CSMC dan atención especializada a personas con trastornos mentales (comunes y severos) y/o problemas psicosociales ^6^. Cuando se recolectaron los datos, entre septiembre de 2021 y marzo 2022, el Perú atravesaba la segunda ola (noviembre 2020 - octubre 2021) y la tercera ola (octubre 2021 - abril 2022) de COVID-19, que trajeron consigo una gran cantidad de contagios y muertes ^15^.

En este artículo, se incluyen a 24 participantes usuarios de los cuatro CSMC, quienes fueron seleccionados mediante muestreo intencional y estratificado ^16^. Los participantes se dividieron en cuatro perfiles: i) usuarios nuevos, ii) usuarios continuadores, iii) familiares de usuarios nuevos y iv) familiares de usuarios continuadores, que se distribuyeron como lo detalla la Tabla 1. Se incluyeron ocho usuarios continuadores (UC) con las siguientes características: diagnóstico de un trastorno mental común (Ej. ansiedad, depresión) o severo (Ej. depresión aguda, bipolaridad, esquizofrenia), mayor de edad y con al menos tres atenciones en los tres meses previos a la declaración del estado de emergencia sanitaria en Perú. Además, se incluyó a cuatro familiares de usuarios continuadores (FUC), mayores de edad y con conocimiento sobre la atención y tratamiento, que su familiar recibía, desde su ingreso al CSMC.

**Tabla 1:** Distribución de los participantes del estudio.

CSMC	Usuarios del CSMC		Familiar del usuario del CSMC		Total por CSMC
	Nuevo	Continuador	Nuevo	Continuador	
CSMC Norte	2	2	1	1	6
CSMC Centro	2	2	1	1	6
CSMC Sur	2	2	1	1	6
CSMC Callao	2	2	1	1	6
Total por perfil	8	8	4	4	24

CSMC: Centro de salud mental comunitaria

Asimismo, se incluyeron ocho usuarios nuevos (UN) con diagnóstico común o severo, mayores de edad y con al menos tres atenciones posteriores al 15 de marzo del 2020 en los CSMC, así como a cuatro familiares de estos usuarios nuevos (FUN), mayores de edad y con conocimiento sobre la atención recibida desde su ingreso al CSMC.

### Técnicas e instrumentos

La recolección de datos se efectuó mediante entrevistas semiestructuradas, para las cuales el equipo de investigación construyó y validó (mediante juicio de expertos) cuatro guías de entrevistas, una para cada perfil (Material suplementario). Estas guías de entrevistas recogían información sobre tres importantes tópicos: el impacto de la pandemia en la salud mental, la experiencia sobre la atención recibida en los CSMC durante la pandemia; y las barreras y facilitadores en la atención, tópicos planteados con el objetivo de entender la relación entre los participantes y los servicios de SM situándonos en el contexto de pandemia, dentro del sistema de salud comunitario. Además, se aplicó una ficha de datos sociodemográficos a cada participante (edad, sexo, diagnóstico y tiempo de atención en el CSMC).

### Procedimientos

Se solicitó autorización a las Direcciones de Salud de las que dependían los CSMC y al jefe del centro; luego los trabajadores de salud ayudaron a identificar e invitar a los usuarios y familiares que cumplían los criterios de inclusión. Quienes mostraron interés fueron contactados telefónicamente para brindarles el consentimiento informado por correo electrónico o WhatsApp, y explicarles los objetivos y procedimientos del estudio. Quienes aceptaron participar brindaron su consentimiento de manera oral, que fue grabado y almacenado, tras lo cual se programaron las entrevistas. Se excluyó a quienes no estaban en capacidad de brindar su consentimiento.

Las entrevistas se realizaron entre septiembre del 2021 y marzo del 2022 por tres investigadoras con experiencia en investigación cualitativa y salud mental. Todas las entrevistas se realizaron en una sola sesión y se realizaron telefónicamente o a través de videollamada en Zoom, tuvieron una duración promedio de 32 minutos y fueron grabadas en audio.

### Análisis de datos

Una vez transcritas, se realizó un análisis temático de las entrevistas ^17^. Inicialmente, dos miembros del equipo (NC y AV) hicieron codificaciones deductivas ^18^ de cuatro entrevistas (una por perfil), tomando como base la guía de entrevista para crear un primer libro de códigos. Posteriormente, cuatro miembros del equipo (NC, AV, VC y RP), desarrollaron la segunda versión del libro de códigos. Éste fue utilizado por dos investigadoras (NC y AV) para hacer una codificación inductiva de cuatro entrevistas, usando el programa ATLAS.ti versión 9 ^19^, en la que lograron más de 95% de acuerdo en el coeficiente de concordancia durante el proceso de codificación de unidades idénticas de análisis lo que permitió identificar la triangulación de información entre los usuarios del CSMC y sus familiares. Finalmente, cada una de las investigadoras (NC, AV) analizó las entrevistas de manera individual, logrando la saturación de datos en todas las categorías de análisis.

La información fue organizada en las categorías y temas detallados en la Tabla 2.

**Tabla 2:** Categorías y temas de análisis según perfil del participante.

Categorías	Temas	Usuarios y familiares continuadores	Usuarios y familiares nuevos
Impacto de la pandemia en la SM	Impacto de las medidas sanitarias	X	X
	Impacto de la suspensión de atenciones presenciales de los CSMC	X	-
Atención de los CSMC	Retorno de las atenciones en SM	X	-
	Motivo de consulta y ruta de acceso al CSMC		X
	Uso de tecnología en los servicios del CSMC antes de pandemia	X	-
	Uso de tecnología en los servicios del CSMC durante la pandemia	X	X
	Servicios ofrecidos por el CSMC durante la pandemia	X	X
	Características de las atenciones antes de la pandemia	X	-
	Características de las atenciones durante la pandemia	X	X
Limitaciones en la atención durante la pandemia	Dificultades en el uso de tecnología	X	X
	Limitaciones en la calidad de la atención	X	X
	Alta demanda de atención	X	X
Facilitadores en la atención durante la pandemia	Labor desempeñada por los trabajadores del CSMC	X	X
	Uso de tecnología en la atención	X	X
Recomendaciones para mejorar la atención en salud mental de los CSMC	Sugerencias de usuarios y familiares para mejorar la atención en los CSMC	X	X

CSMC: Centro de salud mental comunitaria; SM: Salud mental.

### Consideraciones éticas

El estudio fue aprobado por el Comité Institucional de Ética de la Universidad Peruana Cayetano Heredia, en Lima (registro de constancia de aprobación N.° 573-33-20). Todos los participantes dieron su consentimiento informado antes de la entrevista. El equipo realizó un curso de ética y buenas prácticas clínicas.

## RESULTADOS

### Impacto de la pandemia en los usuarios de los CSMC

Entre los usuarios nuevos y continuadores, la COVID-19 se asoció a «muerte», «duelo», «contagios» y «encierro» debido al contexto generado por virus en el país (altas tasas de mortalidad y casos de COVID-19; siendo ello la principal causa de impacto emocional (tristeza, angustia, miedo) en los usuarios. Asimismo, entre los usuarios continuadores, el impacto emocional estuvo también asociado a la preocupación por la continuidad de su atención, principalmente por no saber «cuándo» y «cómo» se retomaría su tratamiento de manera regular.

*Al principio, cuando empezó la pandemia, pues, se quedó todo en el aire, o sea, yo, la verdad es que me quedé sin pastillas al principio, sin citas. [...] cerraron totalmente, yo estaba un poco preocupada por las pastillas* (UC 02, CSMC Lima Norte)

La mayoría de usuarios (nuevos y continuadores) percibieron que la pandemia y la necesidad de adaptarse a las medidas sanitarias exacerbaron los síntomas preexistentes en personas con problemas de salud mental, percepción que fue ratificada por sus familiares, quienes acompañaron a los usuarios como sus cuidadores primarios durante la pandemia.

Con respecto a ello, los familiares de usuarios (nuevos y continuadores) mencionaron que este acompañamiento significó un incremento en la carga de sus actividades, las mismas que estuvieron relacionadas con la supervisión del cumplimento y adherencia al tratamiento (principalmente el farmacológico y las modalidades de atención durante la pandemia), el cumplimiento de la normativas sanitarias (uso de implementos de bioseguridad, aislamiento social, toques de queda, entre otros); y el acompañamiento y soporte socio afectivo a los usuarios. Finalmente, dicho contexto generó un impacto negativo en la salud mental de los familiares.

*(...) Y teníamos que estar encerrados, no salir en la cuarentena. Fue bien caótico, el encierro y eso también deprime, hay cambios en las personas, aún así no tenga un problema psicológico. A todos nos ha afectado de una u otra manera, y con mucha razón al paciente.* (FUC 02, CSMC Callao)

### Atención en los CSMC durante la pandemia

Todos los usuarios continuadores y sus familiares mencionaron que durante la pandemia se suspendieron las actividades presenciales en los CSMC: solicitud de información, programación de citas, atenciones individuales y grupales, y dispensación de medicamentos. Posterior a la declaración del estado de emergencia, los usuarios de los CSMC de Lima Norte, Sur y Centro refirieron que el personal de salud se comunicó telefónicamente para informarles que, en adelante, sus atenciones serían virtuales. No obstante, una usuaria continuadora del CSMC Lima Norte, con quien no se comunicaron telefónicamente, se enteró a través de una conocida de la reanudación del CSMC, exclusivamente para la entrega de medicamentos. Finalmente, los usuarios del CSMC de Callao, al no ser contactados por su CSMC se acercaron presencialmente a solicitar información.

*A partir de marzo, no había nada de citas. Al siguiente mes, recién implantaron las llamadas telefónicas, y que, y que, creo que pasó un tiempo más (...) para que recién salgan las citas telefónicas* (UC 01, CSMC Lima Sur)

En relación a los ocho usuarios nuevos, cinco de ellos no tenían historial previo de atención en salud mental, aunque la mayoría experimentó síntomas anteriormente, mismos que se exacerbaron durante la pandemia. Por esa razón, tras conocer, mediante amigos, familiares o redes sociales, los servicios ofrecidos en los CSMC, decidieron buscar ayuda. Mientras que dos usuarios fueron referidos a un CSMC porque sus establecimientos de salud habituales interrumpieron la atención en salud mental. Finalmente, una mujer fue referida desde la fiscalía por violencia familiar.

*Yo no sabía que me encontraba delicada de salud mentalmente. Cuando un amigo psicólogo viene a visitarme, yo le decía ‘estoy casada’, pero yo soy soltera, osea tenía alucinaciones [...] él me dice: ‘Me parece que tú has hecho un cortocircuito en tu cerebro´ [..] Y que sería bueno que te lleven a un psiquiatra. Entonces me da la dirección del CSMC. Fuí y me atendieron sin problemas* (UN 01, CSMC Lima Sur)

Sobre la programación de las atenciones, los usuarios continuadores refieren que, antes de la pandemia este proceso se realizaba presencialmente. Sin embargo, todos los informantes coincidieron en que, a lo largo de la pandemia, la programación de esta, se efectuó mediante llamadas telefónicas, mismas que sirvieron para corroborar la disponibilidad de citas, fechas, horarios y modalidad de atención.

*Me habían llamado una vez, dos veces… que un día no iba a haber atención porque el doctor no ha venido ...o también haciéndome recordar que tengo que ir mañana… o como es en la noche, me olvido y me recuerdan que mañana a tal hora tengo cita* (UN 01, CSMC Lima Norte)*.*

Con respecto a la atención recibida durante la pandemia, los usuarios coinciden en que el personal del CSMC monitoreaba, sobre todo, su estado emocional y el cumplimiento del tratamiento farmacológico. La mayoría de usuarios y familiares identificaron que la oferta de atención presencial o virtual en los CSMC dependía de la situación de la pandemia (olas de contagio), lo que influía, además de en la modalidad de atención, en su frecuencia, en la prescripción de ciertos medicamentos y la escasez de otros, y en la derivación a servicios específicos como psicología, psiquiatría o medicina, aunado a la percepción de un incremento en la demanda de atención de los servicios del CSMC.

*Me llamaban más seguido para preguntarme cómo iba, cómo estaba, mis emociones, en el caso de la psicóloga. La psiquiatra solamente cada vez que tenían que medicarme [..] La verdad es que en tiempo de pandemia han estado más atentos de cada uno* (UC 01, CSMC Lima Centro).

*Ellos (trabajadores del CSMC) están dándonos la medicina para el usuario, pero como no hay la Olanzapina, eso tenemos que comprarlo yo, porque no hay, estamos yendo al Noguchi para comprar ahí porque es más cómodo* (FUC, CSMC Lima Norte).

En ese sentido, los usuarios continuadores con tratamiento farmacológico, reportaron que al inicio de la pandemia (marzo - mayo del 2020), el primer contacto con sus CSMC fue por vía telefónica y, aunque inicialmente no les ofrecieron retomar sus atenciones individuales, sí pudieron programar el recojo de sus fármacos, y en algunos casos les extendieron la validez de sus recetas para reducir su asistencia al CSMC y evitar exponerlos a posibles contagios. Además, algunos usuarios pudieron recoger sus medicamentos en los CSMC tras recibir, vía WhatsApp, la foto de sus recetas físicas firmadas por el médico tratante.

*El doctor me decía ‘le voy a mandar para 2 meses su medicamento porque ya no puede estar viniendo continuamente’. Entonces, él me decía ‘Usted va a recoger su receta en el centro de salud’. Yo iba con mi DNI, me daban la receta y me daban el medicamento con las indicaciones* (UC 01, CSMC Callao).

Posteriormente, como indican los usuarios nuevos y continuadores, los CSMC priorizaron la atención individual mediante videollamadas a través de Zoom, Meet o WhatsApp. Sin embargo, esta estrategia presentó algunas dificultades descritas en el siguiente apartado, por lo que fue reemplazada por llamadas telefónicas. Unos meses después, entre la primera ola y los primeros meses de la segunda ola (agosto - diciembre del 2020), algunos CSMC de Lima Sur, Centro y Callao retomaron las atenciones presenciales en psicología y psiquiatría. Los usuarios de Callao, mencionaron que sus atenciones duraban hasta 45 minutos, mientras que los usuarios de Lima Sur, Lima Centro y Lima Norte mencionaron que sus atenciones duraron entre 15 y 30 minutos. Los usuarios coincidieron que, en promedio, la frecuencia de atención era cada tres semanas. No obstante, durante el pico de la segunda ola (diciembre del 2020 - abril del 2021), y durante los primeros meses de la tercera ola (enero - marzo del 2022), volvió a priorizarse la atención a distancia, principalmente con llamadas telefónicas, que duraban en promedio 15 minutos y se podían dar cada dos semanas. 

*Hacíamos las citas telefónicas… y él me llamaba (trabajador del CSMC), era una vez al mes que se encontraban conmigo (presencialmente), me preguntaban cómo estaba. Y ya luego me decía: ¿Vas a seguir la misma medicación? Y con esta pastilla, me indicaba, en cualquier momento tenía que yo ir a recoger solamente la receta, nada más, al centro* (UC 01, CSMC Lima Sur).

En general, la mayoría de usuarios continuadores no percibieron diferencias importantes en la duración y la frecuencia de sus atenciones en comparación a antes de la pandemia, además percibieron que el uso de llamadas telefónicas y mensajería por WhatsApp fue transversal a las modalidades de atención durante la pandemia. 

### Dificultades en la atención durante la pandemia

La mayoría de participantes consideraron que la principal dificultad durante la pandemia fue adaptarse a las videollamadas para retomar sus atenciones individuales en los CSMC. Lo cual se puede comprender debido a las siguientes razones descritas por los usuarios: la baja calidad de la señal de Internet en sus domicilios, el gasto que suponía recargar datos de Internet para acceder a las videollamadas, y su desconocimiento en el uso de plataformas como Zoom o Meet. Por estos motivos, los usuarios identificaron que las videollamadas, como modalidad para la atención individual, duraron poco tiempo.

*Como compartía los datos, por ejemplo, mi Internet a veces no funcionaba bien… tuve que hacer un costo adicional para poner Internet Hogar, con más megas, para poder llevar estas sesiones* (UC 01, CSMC Lima Centro).

*Si no podíamos hacer videollamadas, ellos optaban por llamadas telefónicas, tanto el psicólogo como el psiquiatra* (UC 01, CSMC Callao).

Otra dificultad percibida por ambos grupos de usuarios estuvo referida a la calidad de las atenciones virtuales. Haciendo énfasis en su preferencia en las atenciones presenciales, ya que esta modalidad de atención supuso para los usuarios un contacto más directo con sus proveedores; y acceder a las instalaciones de los CSMC para recibir sus consultas, fue relacionado con la posibilidad de contar con un espacio privado e íntimo. En ese sentido, dado que las atenciones virtuales no contaban con estas características fue percibida como una metodología con menor efectividad en su tratamiento.

*Lo que sí [...] un poco es mi tratamiento, ¿no?, porque cuando era presencial era mejor...como que yo sentía que el tratamiento iba funcionando mejor, de manera más… Que evolucionaba de mejor manera. En cambio, virtual no...no, no me parecía* (UC 01, CSMC Lima Centro).

Otra dificultad reportada por los usuarios fue el incremento en la demanda de atención, la cual fue descrita como la principal causa de la saturación de las líneas telefónicas de los CSMC. Esto generó que las citas que se programaban vía telefónica tarden en ser contestadas por el personal del CSMC, o en su defecto, que se agotaran rápidamente, debiendo solicitar citas extras o, en algunos casos, reduciendo la frecuencia de atención. 

*Había muchos pacientes que estaban llamando......me ocupaba toda la mañana. Había unos días que llamaba toda la mañana y contestaban recién cuando llegaba la tarde, y era un poco tedioso* (UC 01, CSMC Lima Sur).

### Facilitadores y beneficios en las atenciones durante la pandemia

Todos los informantes resaltaron que la labor que desempeñó el personal de los CSMC permitió el cumplimiento y la continuidad de su tratamiento. Coincidieron en que, a pesar de que el personal tuvo que enfrentar sus propios retos y exponer su vida para continuar las atenciones, no «desmayaron» en su labor. Además, refirieron que, en todo momento, el personal de los CSMC buscó alternativas para que los usuarios sean atendidos, brindándoles citas extras y/o llamándolos constantemente.

*Te sientes importante, que les importas a ellos, ¿no? En ningún momento, como te digo, ellos desmayaron, no tomaron como pretexto la pandemia para dejar al paciente, de ninguna manera. Al contrario, estuvieron ahí más pendientes que nunca* (UC 01, CSMC Lima Centro).

La mayoría de participantes, resaltó que la incorporación de llamadas telefónicas para monitorearlos, así como para programar y recordar citas, fue beneficiosa y ayudó a los usuarios a sentirse «más acompañados». Finalmente, aunque las líneas estaban frecuentemente saturadas, consideran que programar sus citas por teléfono fue ventajoso, porque evitaban las filas y aglomeraciones dentro del CSMC.

*Se preocupaban (los trabajadores del CSMC) de mi salud y me hacían sentir que era importante* (UC 01, CSMC Callao).

*Pero siempre han estado presentes (trabajadores del CSMC) (...) a mí me gustaba siempre verlas, porque, este, no sé, estaba, necesitaba verlas para poder sentirme bien, siempre me llamaban para programar la cita. Y siempre me llamaban para recordarme* (UC 01, CSMC Lima Centro).

### Recomendaciones para la atención en el CSMC

Al ser consultados sobre sus recomendaciones para mejorar la atención de los CSMC, los usuarios y sus familiares consideraron importante la reapertura de actividades grupales, como el «club psicosocial», un espacio donde los usuarios son acogidos y acompañados en su proceso de integración en la comunidad. Al hacerse las entrevistas, estas actividades se habían retomado, aunque virtualmente, solo en un CSMC. Además, varios usuarios entrevistados mientras predominaban las atenciones virtuales, recomendaron que se retomen las atenciones presenciales. Una tercera recomendación fue que las llamadas telefónicas continuarán, principalmente para monitorear su estado de salud mental, recordarles sus citas y coordinar la entrega de medicamentos. Finalmente, algunos usuarios y familiares recomendaron que se establezcan canales de comunicación entre los usuarios y el CSMC a través de redes sociales.

## DISCUSIÓN

Nuestro estudio describe las experiencias de los usuarios de los CSMC y de sus familiares en relación a la atención de salud mental ofrecida en CSMC durante la pandemia.

Los participantes perciben un agravamiento del malestar emocional entre los usuarios, vinculado a las limitaciones para realizar sus actividades cotidianas y a la incertidumbre sobre la continuidad de su atención, pero además al temor por contagiarse de la COVID-19, o perder algún familiar a causa de esta enfermedad. El marco de estas experiencias son las estrictas restricciones que se implementaron en el Perú, como los «toques de queda» ^20^, pero además la alta mortalidad de la COVID-19, durante largo tiempo, siendo una de las más altas del mundo ^15^. En línea con nuestros resultados, un estudio en China, con pacientes no psiquiátricos y psiquiátricos, concluyó que los segundos presentaban síntomas más elevados de estrés, depresión, ansiedad, e insomnio en la pandemia, y más preocupaciones sobre su salud física, irritabilidad e ideación suicida durante los meses con más restricciones ^21^.

Además, en este contexto, los familiares de los usuarios experimentaron un aumento de su carga de trabajo y desgaste emocional, lo que coincide con un estudio realizado en México, que evidenció que la carga de los cuidadores de personas con discapacidad, especialmente con enfermedades crónicas, se agudizó durante la pandemia ^22^.

Un resultado importante es la identificación de cambios en la atención ofrecida por los CSMC durante la pandemia. En el Perú se interrumpió las atenciones presenciales en salud mental frente a la urgencia de la COVID-19, al igual que mayoría de países del mundo ^23^. Así, si bien las normativas peruanas priorizaban la atención en salud mental, no contemplaban dicha atención en medio de una emergencia sanitaria, por lo que fue necesario crear lineamientos para garantizar esta atención utilizando tecnología ^6^. Algo similar se evidenció en el 70% de países del mundo, que adoptaron la telemedicina como principal vía para proveer atenciones en salud mental durante la pandemia ^24^.

En el Perú; sin embargo, el sistema de salud no cuenta con el equipamiento y tecnología adecuada, por lo que muchos usuarios carecen de equipos, servicios y recursos para acceder a la telemedicina, lo que limitó su uso fundamentalmente a llamadas telefónicas y mensajes desde los CSMC para programar las atenciones individuales virtuales o presenciales, recordatorio de citas y monitorear el estado de salud de los usuarios. Aun así, las llamadas telefónicas fueron una estrategia factible y efectiva; además, fueron bien recibidas por los usuarios en Perú. Contexto similar, se evidenció en México, en donde las videollamadas fueron también, en ocasiones, reemplazadas por llamadas telefónicas, las mismas que resultaban más accesibles para los usuarios ^24^. El uso de llamadas se dio también en países ricos, como Australia, donde se usaron para realizar consultas remotas de enfermería, psiquiatría, psicología y trabajo social, con buena aceptación de los usuarios ^25^.

Nuestros hallazgos revelan la preferencia de las atenciones presenciales sobre las virtuales**.** La evidencia internacional sobre la aceptación de servicios de telemedicina es heterogénea. Por un lado, se considera que puede afectar el vínculo terapéutico entre usuario y proveedor, y dificulta la evaluación de ciertos signos y síntomas, mientras que, por otro lado, ofrece continuidad a la atención en salud mental ^26^. Entre los usuarios de los CSMC, la menor preferencia por las atenciones virtuales podría deberse, en parte, a las dificultades que enfrentaron para tener una adecuada conexión de Internet durante las consultas remotas.

A nivel del sistema de salud, es importante resaltar que la implementación de estrategias tecnológicas requiere de inversión adicional, aunque según la Organización Mundial de la Salud solo el 17% de los países contó con financiación extra para los requerimientos en la implementación de telemedicina durante la pandemia ^23^. En el Perú, si bien existió un mayor presupuesto asignado para los servicios de salud mental, la información disponible no revela si este financiamiento se destinó a dotar de equipamiento e Internet a los CSMC y entrenar al personal, estrategias recomendadas por los lineamientos internacionales ^26^.

El uso de la tecnología en la pandemia permitió dar continuidad al tratamiento de los usuarios de los CSMC, pero enfrentó varias limitaciones. Al ser la salud mental una prioridad del Estado, pero además un aspecto esencial en emergencias como la pandemia, el sistema de salud debe invertir en equipos y servicios de telefonía e Internet, así como en entrenamiento, que garantice que prestadores, usuarios y familiares pueden ofrecer y recibir atenciones remotas sin dificultades. La evidencia revela que, si bien la telemedicina supone una reestructuración del sistema y una inversión económica al inicio, los servicios de telemedicina en salud mental resultan ser costoefectivos a mediano plazo ^27^.

Además, dado que el uso de llamadas telefónicas para programar y recordar citas, así como monitorear síntomas y tratamientos fue positivamente valorado y ha mostrado buenos resultados en otros países ^25^, debería mantenerse y profundizarse en los CSMC. Se podrían, por ejemplo, añadir llamadas para monitorear el estado emocional, con preguntas estandarizadas, como otros estudios recomiendan ^28^.

Asimismo, tomando en cuenta la sobrecarga que la pandemia supuso para los cuidadores, resulta importante ofrecerles tanto orientación para acompañar a los usuarios como soporte emocional para protegerlos y permitirles continuar con su labor de cuidado ^29^.

En relación a las limitaciones del estudio, el grupo investigador, tuvo que orientar virtualmente a usuarios a resolver problemas con el uso de Zoom, y en algunos casos, se tuvieron que adaptar las videollamadas a llamadas telefónicas, debido a los recursos de los usuarios. Las entrevistas se realizaron en distintos momentos, durante la segunda y tercera ola de la pandemia, por lo que las experiencias de los participantes podrían diferir, en parte, por el contexto. A eso podrían sumarse algunos sesgos de recuerdo por el tiempo transcurrido desde el inicio de la pandemia. Sin embargo, las entrevistas se realizaron con una guía semiestructurada que permitía guiar al participante a través de los distintos momentos de su atención en salud mental. Una fortaleza del estudio fue contar con la participación de familiares de los usuarios (nuevos y continuadores), esto permitió que la información recolectada tenga un mayor respaldo.

En conclusión este estudio que recogió las narrativas de diferentes actores (pacientes nuevos, continuadores y sus familiares) encontró que la pandemia de la COVID-19, las restricciones sanitarias y sociales, y el cierre de establecimientos de salud mental, exacerbaron los síntomas de las personas con problemas de salud mental y aumentó la carga emocional, física y social en los cuidadores. Tras suspenderse las citas presenciales, la atención en los CSMC se retomó con apoyo de la tecnología, principalmente de llamadas telefónicas y mensajería móvil que sirvieron para efectuar atenciones individuales, programar y recordar citas, y para monitorear el estado emocional y tratamiento de los usuarios. Las limitaciones descritas por los usuarios en sus atenciones fueron: el incremento en la demanda de atención, dificultades para acceder a videollamadas, y una percepción de que las atenciones virtuales tenían un menor impacto en su recuperación. Sin embargo, también se identificaron facilitadores para la atención, como el compromiso del personal de salud y el uso frecuente de llamadas telefónicas, que permitieron a los usuarios sentirse acompañados durante el contexto de pandemia.
